# The Influence of Sense of Place on Elementary School Students’ Creativity During the COVID-19 Pandemic: The Mediating and Buffering Effects of Psychological Resilience

**DOI:** 10.3389/fpsyg.2022.775624

**Published:** 2022-04-26

**Authors:** Yanhua Xu, Qiaoling Wang, Dongmei Zhang, Peiying Lin

**Affiliations:** ^1^College of Resource Environment and Tourism, Capital Normal University, Beijing, China; ^2^Beijing Academy of Educational Sciences, Beijing, China; ^3^College of Teacher Education, Capital Normal University, Beijing, China

**Keywords:** sense of place, creativity, psychological resilience, mediating and buffering effects, COVID-19

## Abstract

**Purpose:**

To understand the relationship between sense of place and creativity and the mechanisms that affect this relationship, the researchers constructed a mediation model to examine the effect of sense of place on creativity and the mediating role of psychological resilience in elementary school students during the COVID-19 pandemic.

**Methods:**

A total of 1,711 students in an elementary school in Beijing, China, were surveyed using Chinese-language versions of the Sense of Place Scale, the Psychological Resilience Scale-Short Version, and the Innovative Behavior Inventory. SPSS (version 23) and PROCESS plug-in (version 3.3) were used for correlation and mediation analyses.

**Results:**

(1) Correlation analysis revealed that sense of place was positively related to psychological resilience (*r* = 0.445, *p* < 0.01) and creativity (*r* = 0.590, *p* < 0.01). (2) Psychological resilience was also positively correlated with creativity (*r* = 0.625, *p* < 0.01). (3) Further, after controlling for gender and grade level, it was found that sense of place directly predicted creativity and that sense of place also indirectly predicted creativity through psychological resilience. The direct effect (0.45) and the mediating effect (0.23) accounted for 65.95 and 34.05% of the total effect, respectively.

**Conclusion:**

The results demonstrate that sense of place is a positive predictor of creativity and can play a facilitating role to some extent. Moreover, psychological resilience is a mediating factor, acting as a buffer between sense of place and creativity. These results contribute to a more comprehensive understanding of the mechanisms influencing creativity.

## Introduction

The novel COVID-19 has had a dramatic impact on humans (Zhao et al., 2020). This global public-health emergency is likely to persist for a long time (Zhou et al., 2020). As the epidemic developed, the government of Chinese, like those of other countries, adopted quarantine in the home and mask-wearing outside the home as the core means of reducing widespread interpersonal transmission of the virus (Wang et al., 2020). As a result of these prevention and control measures, the student population presented with varying degrees of anxiety and depression during the acute epidemic phase for reasons such as financial stress, academic delays, impact on daily life, and shortage of social support (Dhar et al., 2020). These conditions have deeply affected the mental health of students, and therefore, it is crucial to study how the COVID-19 pandemic influenced them psychologically.

*Psychological resilience* is a factor that encompasses many aspects of students’ ability to change and to recover and maintain their mental health in the face of changes brought about by an unexpected event. It is the ability or dynamic process by which an individual is able to adapt and continue to develop after a serious threat (Huntsinger and Ray, 2016). Past research has shown that psychological resilience can buffer against psychological trauma from public-health events (Ran et al., 2020), terrorist attacks (Zeidner and Kampler, 2020), cancer (Mystakidou et al., 2015), depression (Howell et al., 2020), and negative emotions (Abiola and Udofia, 2011), and it can help people reduce their vulnerability to challenges and difficulties in their work environment (Howell et al., 2020). However, few studies have examined the mechanism of action of psychological resilience as an outcome variable in elementary school student populations during the COVID-19 pandemic. Therefore, it may be important to study in depth how the psychological resilience of elementary school students has influenced and been influenced during the COVID-19 pandemic.

In addition, *creativity* may also be stimulated during the COVID-19 pandemic, as exemplified by the 4C model of creativity (Kapoor and Kaufman, 2020). The 4 Cs which consisted of Mini C, Little C, Pro C, and Big C were proposed by Kaufman and Beghetto in 2009. Mini C is considered as novel, individualized, and meaningful interpretations of experiences, behaviors, and events; Little C, also known as everyday creativity, encompasses all walks of life of ordinary people; Pro C refers mainly to the professionalism demonstrated by creative people in the relevant fields; and Big C, also known as outstanding creativity, generally refers to the creativity exhibited by great people or people with extraordinary achievements. For instance, people might generate a variety of novel or useful ideas to distract themselves from or find fun in their boring isolated life during the COVID-19 pandemic, which is the concentration of Mini C and Little C. Still, schools at all levels transformed traditional offline classes into online courses during the COVID-19 pandemic, which is a manifestation of Pro C. In addition, Pro C is more prominent in scientific creativity. A case in point is that researchers have begun developing a vaccine against this novel coronavirus since its genetic sequence was detected in February 2020 after the outbreak of COVID-19 pandemic (Ren et al., 2020). Further, since the COVID-19 pandemic began spreading around the world, clinical trials for this vaccine have been underway; researchers have shifted their agenda from developing a MERS vaccine to developing a COVID-19 vaccine and have thus been motivated to get ahead of the race with this novel virus (Masetti et al., 2020). This is often referred to as the Big C. However, under the context of the COVID-19 pandemic, few studies are focused on the mechanism of action with creativity as an outcome variable among the population of elementary school students. Therefore, it may also be important to delve into how the situation of the COVID-19 pandemic is influenced by the creativity of this particular population.

The existing literature offers many studies related to psychological resilience (e.g., Marx et al., 2017; Liu et al., 2020; Xu et al., 2021). Its relationship to creativity has also been of interest to researchers (Marwa and Milner, 2013; Chen, 2015; Anser et al., 2020; Xu et al., 2021). In one study, it was mentioned that artistic ethnographic innovation best begins with the experience of a sense of place, one of the conjunctions of environment and sensation (Kapoor and Kaufman, 2020). Further, this also shows the importance of the sense of place, which is one of the combinations of environment and sensation, for creativity. Other factors have also been found to be related to creativity, including environment (e.g., Richards, 1992; Cole et al., 1999; Walter, 2012; Pugsley and Acar, 2020), senses (e.g., Chessick, 1996; Berg and Hallberg, 1999; Fraser et al., 2021), and emotions (e.g., Kopcso and Lang, 2017; Demetriou and Nicholl, 2021) have been found to be related to creativity. However, few studies have focused on the relationship between sense of place—the combination of environment and perception/emotion—and creativity, the relationship between sense of place and psychological resilience, and the possible relationship between sense of place, psychological resilience, and creativity.

To enhance recognition of elementary school students’ creativity during the COVID-19 epidemic, this study explored the relationship between sense of place and creativity and also the mediating role of psychological resilience. In the next section, the definitions of these three variables, the influencing variables, and the relationships among them are presented. Two frequently reported factors that might have influenced students’ psychological resilience and creativity—gender and grade level—were considered as covariates and controlled for in the process.

## Theoretical Basis and Hypothesis

### Sense of Place

Since the 1970s, scholars have gradually developed the concept of sense of place in the face of the loss and blurring of local identity brought about with globalization (Changhu, 2020). Lowenthal and Tuan (1975) were the first to propose the concept of *sense of place*, which they believed encompasses both the inherent characteristics of a place and people’s sense of attachment to that place. Zhu and Liu (2011) maintained that the sense of place is mainly shown by the objective materials and social functions of a locality, i.e., its meaning, and that it creates an emotional link between human beings and the place. It is also a response to the gradual integration of the self into the environment through human-place interactions. It is influenced by personal emotional integration, values, and other emotional factors. According to Steele and Steele (1981), human sense of place is an outcome of the interaction between people and place, an experience acquired by people with respect to place. The concept has been widely used in management, sociology, psychology, and architecture.

There remains no consensus on the definition of sense of place. For instance, Tuan creatively proposed *topophilia*, which implies that humans have always experienced and experimented with their environment through their sense and unique experience, a process in which they develop a sense of place (as cited in Tapsuwan et al., 2011). Steele and Jackoson pointed out that the sense of place is a sense of belonging that is constructed along with a sense of time (as cited in Steele and Steele, 1981), and Massey (1994) proposed that the sense of place is a dynamic concept that changes over time. According to existing literatures, the sense of place is a multidimensional complex structure (Lowenthal and Tuan, 1975; Jorgensen and Stedman, 2001; Tapsuwan et al., 2011; Changhu, 2020). Scholarly analysis of its intrinsic elements has produced different perspectives (Lowenthal and Tuan, 1975; Jorgensen and Stedman, 2001; Tapsuwan et al., 2011; Changhu, 2020). It has been classified as having two to six dimensions by scholars (Zhu and Liu, 2011; Juan, 2014; Tingting and Zhao, 2015). The most influential one is the Sense of Place Scale proposed by Jorgensen and Stedman (2001). This scale, represented by a 12-item scale ranging from low to high, quantifies the sense of place as having three dimensions (place identity, place attachment, and place dependence).

### Creativity

The term “creativity” was first defined by American psychologist Guilford (1950) as a thinking and behavioral process that generates a valuable and original idea, product, or solution. Since the term was proposed, the connotation and theoretical basis of creativity have been continuously enriched and expanded (Plucker et al., 2004; Xu et al., 2021). However, to date, there is still no universally accepted academic definition of creativity, except for a generally accepted view that creativity is a cognitive process that is characterized as the ability to generate novel ideas (Runco and Basadur, 1990; Xu et al., 2021). Such a cognitive process has long been shown to be closely related to intelligence (Sternberg and Lubart, 1991), e.g., to the breadth of attention (Fredrickson and Branigan, 2005), creative problem solving (Isen et al., 1987), and divergent thinking (Zhu et al., 2019).

As mentioned earlier, the sense of place is an environmental and perceptual complex like that, and when it is related to creativity, the theoretical framework is considered in relation to the following theories of creativity. The first is Rhodes' (1961) proposal of the *4Ps model of creativity* which integrates people, process, product, and environment. The second is Amabile’s (1988) model of the components of creativity, which takes into account cognitive, individual, motivational, and social factors and includes domain-relevant skills, creativity-relevant skills, and task motivation. Later, he enriched the model by adding a “social environment component” to it (Amabile, 1996). The third is the theory of *creativity investment* proposed by Sternberg and Lubart (1991), which states that intelligence, knowledge, thinking style, personal traits, motivation, and environment may influence creativity, and so creativity is the result of a mix of individual psychological mechanisms and environmental factors. In addition, at the beginning of the 21st Century, Plucker et al. (2004), after reviewing 90 influential journal articles, proposed that creativity is the result of the interaction among ability, process, and environment, and that individuals or groups have the potential to produce novel and useful works. In Kaufman and Beghetto (2009) proposed the *4C model* of creativity performance, namely, “Big C” (the creative capability of an outstanding, eminent person), “Pro C” (the innovative capability of a professional in a certain field), “Little C” (innovation in everyday life), and “Mini C” (experiences, activities, and events that are fresh to an individual).

All of the above theories fit, to a greater or smaller extent, with the focus of this study on the relationship between elementary school students’ sense of place and creativity. However, the theory of creativity investment and the 4C model fit better. Therefore, they lay a theoretical foundation for exploring the relationship between elementary school students’ sense of place and creativity in this study.

According to the above-mentioned theories, clearly, several recent studies have been dedicated to discussing the factors influencing creativity, such as motivation, cognition, sense, perception, emotion, and environment (Cole et al., 1999; Edl et al., 2014; Du et al., 2020; Xu et al., 2021). The main body of this research provides a broader interpretation of what creativity is and what processes are involved. It can be inferred that creativity is influenced by many factors, and this inference agrees with the results of these current studies (e.g., Isen et al., 1987; Runco and Basadur, 1990; Fredrickson and Branigan, 2005; Xu et al., 2021). A number of recent studies have been devoted to discussing factors influencing creativity, such as motivation, cognition, sense, perception, emotions, and environment (e.g., Cole et al., 1999; Edl et al., 2014; Du et al., 2020; Xu et al., 2021). Different contexts, emotions, and feelings have been shown to affect creative performance differently, e.g., corporate innovation (Ding et al., 2019), artistic creativity (Xurui et al., 2018), and everyday emotional creativity (Han et al., 2019), but these still come down to cognitive processes in essence (Leschziner and Brett, 2019). According to Janssen (2000), creativity in this study is considered as the process through which people promote and practice creative ideas as they are generated. It consists of three stages: idea generation, idea promotion, and idea practice.

Among the available literatures, based on the theoretical framework of creativity investment, studies on the relationship between creativity and environment (e.g., Cole et al., 1999; Dul and Ceylan, 2011; Gong et al., 2019), sense (e.g., Chessick, 1996; Berg and Hallberg, 1999; Fraser et al., 2021), and the interaction between creativity and emotion (e.g., Kopcso and Lang, 2017; Mastria et al., 2019; Xu et al., 2021) have also received much attention. One mainstream consensus among many studies is that a favorable environment promotes creativity (e.g., Cole et al., 1999; Dul and Ceylan, 2011; Gong et al., 2019). Another mainstream view is that positive emotions and perceptions promote creativity (e.g., Rouff, 1975; Humke and Schaefer, 1996; Yawen et al., 2021).

Despite differences in perspective, all conceptualizations share the commonality that the sense of place is a mix of environment and perception (Zhu and Liu, 2011; Juan, 2014; Tingting and Zhao, 2015). Currently, few studies have investigated into the relationship between sense of place and creativity; however, some researchers have focused on the relationship between environment and creativity (e.g., Dul and Ceylan, 2011; Gong et al., 2019; Pugsley and Acar, 2020), even arriving at the conclusion that environment is positively correlated with creativity (Richards, 1992; Dul and Ceylan, 2011; Pugsley and Acar, 2020); i.e., the more positive the environment, the higher the level of creativity. In addition, some studies have reported on the relationship between perception (or emotion) and creativity (e.g., Chessick, 1996; Humke and Schaefer, 1996; Rui et al., 2021). Some studies have also found that positive emotional effect is sometimes positively correlated with creativity (e.g., Humke and Schaefer, 1996; Zhuravlyova and Zhuravlyov, 2015). Given that sense of place is a mix of environment and perception (or emotion), these studies help understand the relationship between sense of place and creativity and the relationship among environment, perception (or emotion), and creativity. There were also some explorations on the 4C model during the COVID-19 pandemic. In other words, sense of place may be positively correlated with creativity. This positive correlation may be manifested as: the higher the sense of place, the higher the level of Mini C or Little C (in writing science and art, etc.) of elementary school students. Therefore, we hypothesize that,

*H1*: Sense of place has a positive predictive effect on creativity.

### Psychological Resilience

In the 1970s, psychologists found that sequelae were reported in children who had been abused, but that these consequences were mild (Mrazek and Mrazek, 1987). Scholars attributed this result to the presence of psychological resilience (Luthar et al., 2000). Among other things, *resilience* refers to positive adaptation in the face of stress or trauma (Block and Kremen, 1996; Tugade and Fredrickson, 2004; Xu et al., 2021). Resilience is also seen as a personality trait with a stress-resistant attitude that influences the process of regulation of emotions (Xu et al., 2021). There are also many theories about what psychological resilience is. For example, it has been argued that *psychological resilience* is the ability or dynamic process by which an individual is able to maintain good development even after being exposed to serious threats (Hou and Ng, 2014; Xu et al., 2021). In normal field of developmental psychology, for example, psychological resilience is considered to be the ability of people to react and respond well to events or experiences that may have a significant impact on them (Huisman et al., 2017; Xu et al., 2021). It is a dynamic process that involves senses, emotions, and cognition (Block and Kremen, 1996; Tugade and Fredrickson, 2004). Psychological resilience enables individuals to find the positive in the negative and to adapt and bounce back from the changing external environment (van Riper et al., 2013). Psychological resilience can be divided into individual resilience and collective resilience (Xu et al., 2021). From a behavioral point of view, psychological resilience is seen as a set of attitudes and skills to deal with negative emotions (van der Werff et al., 2013). From these various definitions, it can be concluded that psychological resilience refers to an individual’s ability to recover from negative experiences and to adapt to the changing external environment (Campbell-Sills and Stein, 2007).

Rutter (1985) pointed out that protective factors can influence and change a person’s response to hazards in the environment, but he also emphasized that they are not the same as positive experiences (Rutter, 1985). Protective factors may comprise experience, the individual’s own qualities, perception (or emotion), and the surrounding environment (Rutter, 1985; Mrazek and Mrazek, 1987).

Currently, several theoretical models of protective factors of psychological resilience exist. For example, Mandleco (2000) proposed a systematic model of children’s psychological resilience based on an analysis of previous studies. Also, a dynamic model of psychological resilience was jointly developed and proposed by several scientific institutions in the United States (Mandleco, 2000). Both models divide the protective factors for psychological resilience into two types, internal and external (Zeng et al., 2021). Internal factors include biological factors, such as physical condition, temperament, and gender, and psychological factors, such as intelligence, cognitive style, problem-solving skills, and personality. External factors include environmental factors, such as family, society, and school (Zeng et al., 2021).

Few studies have investigated the relationship between sense of place and psychological resilience. However, one study showed that a positive educational environment can increase psychological resilience in medical students (Xu et al., 2021). Another study found a positive correlation among subjective wellbeing, a perception (or emotion), and psychological resilience (Can, 2020). Given that sense of place is a combination of environment and perception (or emotion) and that there is more overlap between it and environment and perception (or emotion), it is highly likely that sense of place is positively correlated with psychological resilience. This leads to the following hypothesis.

*H2*: Sense of place has a positive predictive effect on psychological resilience.

Related research has shown that psychological resilience is positively correlated with creativity (Xu et al., 2021). In education, psychological resilience may give learners a strong ability to challenge their environment, which in turn stimulates their positive emotional affect and their motivation to learn (e.g., Chen and Padilla, 2019; Xu et al., 2021). Luthans et al.’s (2006) research found that resilience at work may produce an internal mechanism of adaptation, persistence, or transcendence, which in turn allows individuals to maintain their creativity through steady effort. The following hypothesis emerged from a literature review that addressed this theme.

*H3*: Psychological resilience has a positive predictive effect on creativity.

Based on the existing literature and the three hypotheses above, we further propose the following hypothesis.

*H4*: Psychological resilience plays a mediating and buffering role between a sense of place and creativity.

**Figure 1:**
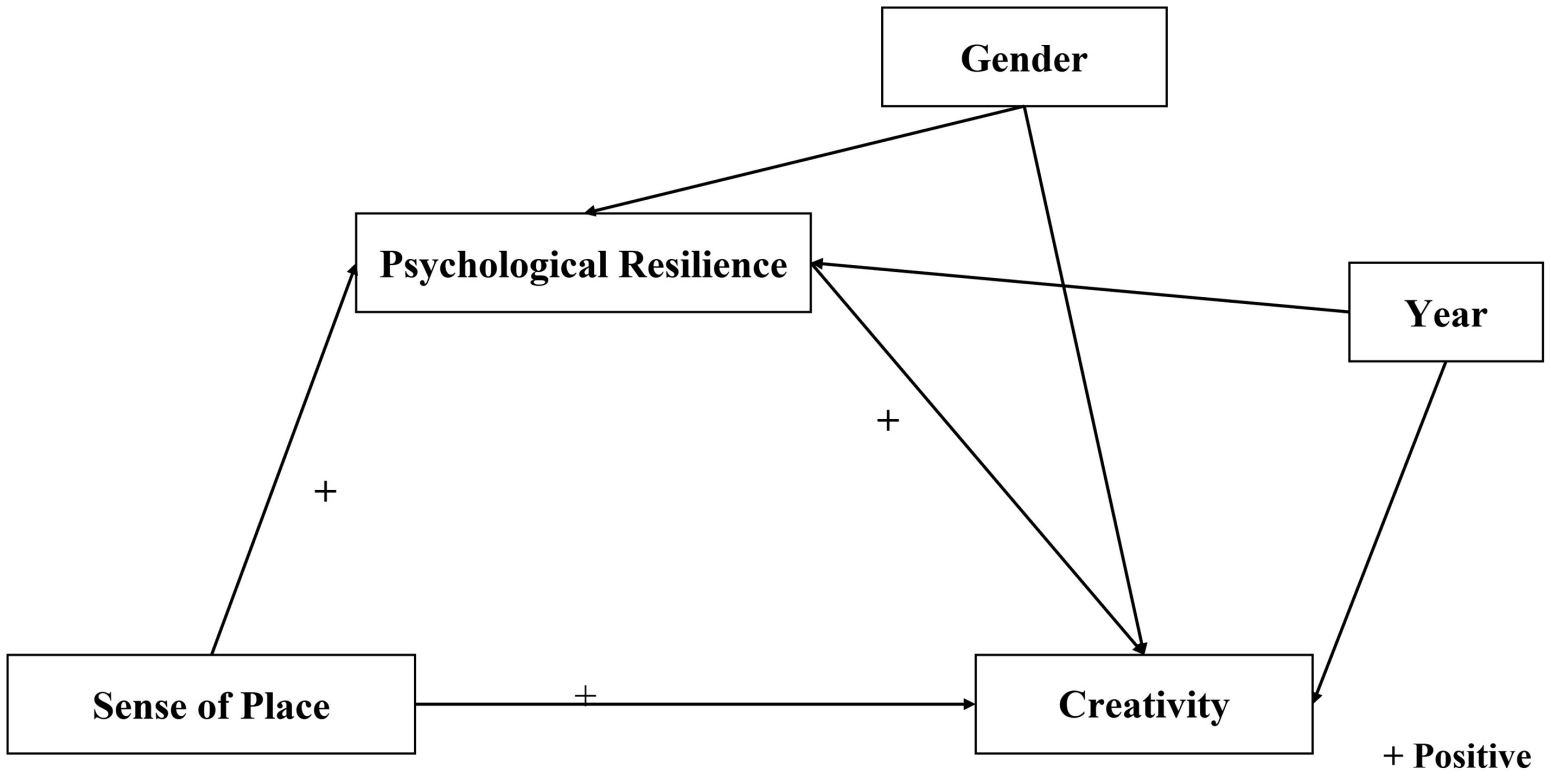
The relationships examined in the study.

Figure 1 shows a graphic of the mediation model proposed in the four hypotheses and depicts the relationship between the independent variable, the mediating variable, the dependent variable, and the two covariates.

## Materials and Methods

### Participants and Procedures

This study was conducted in an elementary school in Beijing, China. Of the 1,800 students in this school, 1,711 participated by filling out the questionnaire. After the data collection was completed, the researchers checked the validity of the completed questionnaires, and the actual number of valid questionnaires was 1,711. Among them, 914 (53.4%) were completed by males and 797 (46.6%) by females. By academic level, 346 (20.2) were in first year, 329 (19.2%) in second year, 316 (18.5%) in third year, 251 (14.7%) in fourth year, 257 (15.0%) in fifth year, and 212 (12.4%) in sixth year. Prior to finalizing the study design, researchers had conducted exploratory focus-group interviews with students at the school to clarify the emotional characteristics and psychological states of the students. The majority of respondents reported that they were in low spirits during the COVID-19 pandemic. However, their identification and attachment to Beijing and to their school gave them an inner urge to alleviate and recover from this dejection.

This study used a correlational design with an online questionnaire as the data collection method. The questionnaires were filled out between 10 June and 15 July 2020. At the end of a school day, classroom teachers showed students the QR code that linked to the questionnaire. Students who agreed to complete the questionnaire scanned the QR code, entered the fill-in screen, answered the questions, and then clicked Submit (“QR” is an abbreviation for “quick response,” and a QR code is a readable barcode that contains a lot of information. A device such as a cell phone or tablet scans the QR code with a camera, recognizes the binary data, and opens a specific link). In China, QR codes are widely used to open specific link interfaces and various applications such as those used for financial payments, personal identification, and information queries. The QR code in this study linked to the webpage where participants could complete the study questionnaire. It should be emphasized that before scanning the code, the classroom teachers briefed in detail the parents and students about the purpose of the study. Before the students filled out the questionnaire, the study was explained to their parents and consent was obtained from the parents.

### Materials

The questionnaire used in this study consisted of four sections and included 33 items: demographic information, Sense of Place Scale, Psychological Resilience Scale, and Innovative Behavior Inventory. Demographic information included gender and academic level. The Sense of Place Scale, Psychological Resilience Scale, and Creative Behavior Inventory were originally developed in English and translated into Chinese for this study. In order to improve the quality of translation, the back-translation method (Brislin, 1970) was used as: the first researcher translated the English scales into Chinese, then the second researcher back-translated the Chinese version into English, and finally the third researcher compared the original, translated, and back-translated versions of the scales to assess the accuracy of the translation. The translation was corrected and optimized before the questionnaire was finalized, which should have ensured the equivalence of the Chinese- and English-language scales.

### Sense of Place Scale

This study used the Sense of Place Scale developed by Jorgensen and Stedman (2001). The scale consists of 12 items, and after discussion, some of the wording was modified to fit the language and life experiences of elementary school students. The scale includes place identity (e.g., “The school and the neighborhood where I live are relevant to me and a reflection of my existence” and “The school and the neighborhood where I live are closely related I can be my true self”), place attachment (e.g., “The school and the neighborhood I live in relax me” and “The school and the neighborhood I live in make me happy”), and place dependence (e.g., “When I do the things I like best, I am happy,” “When I do my favorite things, I like school and the neighborhood I live in the most,” and “When I do my favorite things, there is no substitute for school and the neighborhood I live in”). The scale assesses participants’ feelings, responses, and agreement on a 5-point scale (1 = completely disagree; 5 = completely agree). The possible scores for participants ranged from 12 to 60 in this study; the internal consistency coefficient of the scale was 0.792.

### Psychological Resilience Scale-Short Version

This study used the Psychological Resilience Scale-Short Version (10-item Connor-Davidson Resilience Scale or CD-RISC-10) as modified by Campbell-Sills and Stein (2007). The original scale consisted of 10 items (e.g., “I can adapt flexibly when changes occur” and “I can respond with humor when faced with problems”). Seven additional items were added to the scale that took into account the actual situation of elementary school students during the COVID-19 pandemic for a total 17 items. To maintain congruency with the two other scales, the original 4-point rating scale was converted to a 5-point scale, ranging from 1 = completely disagree to 5 = completely agree. The score conversion resulted in a range of possible student scores from 17 to 85. In this study, the internal consistency of the scale was 0.848.

### Innovative Behavior Inventory

This study used the Innovative Behavior Inventory, which was developed by Janssen (2000) by integrating the conceptualizations several scholars. The scale contains 9 items, and after discussion, some of the wording was modified to fit the language and life experiences of elementary school students. The scale measures the innovative-idea generation stage (e.g., “I often generate new ideas when I encounter difficulties” and “I seek new methods, techniques, or tools”), the idea promotion stage (e.g., “I seek support from my teacher for innovative ideas” and “I facilitate the flow of innovative ideas within my organization”), and the diffusion stage (e.g., “I will translate innovative ideas into achievable practices” and “I will evaluate the benefits of innovative ideas”). The range was from 1 (= almost always not true) to 5 (= almost always true), and participants’ possible scores ranged from 9 to 45. In this study, the internal consistency of the scale was 0.954.

### Data Analysis

In this study, SPSS 23.0 was used to process and analyze the data. Since the study used self-report for data collection, the common method biases were tested using the Harman one-way test before data processing (Podsakoff et al., 2003) to ensure the validity of the data. The test was conducted by examining 31 items of the questionnaire related to three variables. The results showed that five factors had eigenvalues greater than 1. The contribution of the five factors to the total variance was 64.151%, and the variance explained by the first factor was only 36.969%, which did not reach the critical criterion of 40% (Hao and Long, 2004). Therefore, there is no significant common method bias in this study.

We then performed descriptive analysis, correlation analysis, and model testing of the data based on the research hypotheses. First, we examined the concentration and dispersion trends of the data through descriptive analysis. Then, we analyzed correlations among the variables to test the relationships among the independent, mediating, dependent, and moderating variables by calculating Pearson correlation coefficients. Based on the correlations, the research hypotheses proposed in this paper were further examined by using the PROCESS (version 3.3) plug-in SPSS to test the model’s mediating effects [The PROCESS plug-in was developed by Hayes specifically for path analysis-based moderating and mediating analyses and combinations thereof (Bolin and Hayes, 2014)].

## Results

### Descriptive Statistics and Correlation Analyses

Descriptive statistics and Pearson matrix correlation coefficients were calculated using SPSS 23.0 to analyze the mean, standard deviation, and correlation coefficients for sense of place, psychological resilience, and creativity of elementary school students. The results of descriptive statistical analysis are shown in Table 1, and the results of Pearson matrix correlation coefficients are shown in Table 2.

**Table 1:** Descriptive statistics for the three variables.

Variables	*N*	Mean	SD
Sense of place	1,711	41.47	7.344
Male	914	41.54	7.344
Female	797	41.39	7.348
Year			
1	346	41.37	7.595
2	329	41.86	6.854
3	316	41.56	7.335
4	251	41.7	7.492
5	257	40.82	7.369
6	212	41.46	7.499
Psychological resilience	1,711	62.87	9.774
Male	914	62.79	9.919
Female	797	62.96	9.61
Year			
1	346	62.24	10.211
2	329	62.62	10.004
3	316	62.26	9.181
4	251	63.79	9.967
5	257	63.42	9.268
6	212	63.42	9.872
Creativity	1,711	31.22	8.418
Male	914	31.59	8.444
Female	797	30.8	8.372
Year			
1	346	30.92	8.65
2	329	31.06	8.368
3	316	30.27	8.411
4	251	31.88	8.448
5	257	31.38	7.651
6	212	32.41	8.849

**Table 2:** Pearson’s *r* for the three variables.

S. No.	Variables	Sense of place	Creativity	Psychological resilience
1.	Sense of place	–		
2.	Creativity	0.59^**^	–	
3.	Psychological resilience	0.45^**^	0.63^**^	–

* *p* < 0.05; ^**^p < 0.01.

As shown in Table 2, sense of place was significantly and positively correlated with general creativity (*r* = 0.59, *p* < 0.01) and psychological resilience (*r* = 0.45, *p* < 0.01). Psychological resilience was associated with creativity (*r* = 0.63, *p* < 0.01). The results of the correlation analysis initially supported the subsequent test of mediating effects.

### Mediation Analysis

The PROCESS plug-in (version 3.3) was used to conduct a mediation analysis with sense of place as the independent variable, creativity as the dependent variable, and psychological resilience as the mediating variable (model #4). Based on the literature review, the three most commonly reported factors that influenced the dependent and mediating variables—gender and grade level—were considered as covariates in this study. Both were transformed into dummy variables before being entered into the mediating model as covariates.

**Figure 2:**
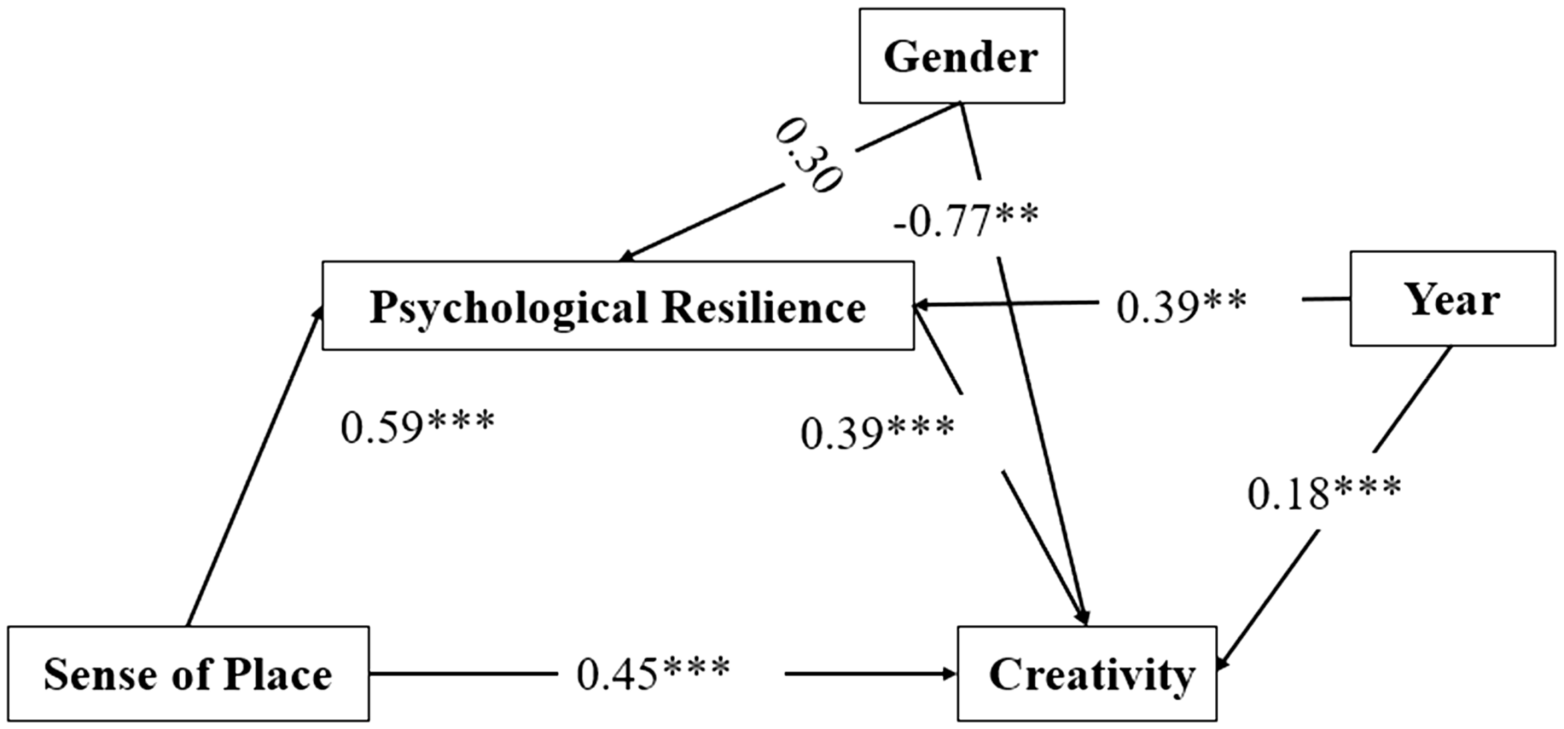
The mediation model showing relationships between sense of place and creativity and the mediating role of psychological resilience. *p < 0.05; **p < 0.01; ***p < 0.001.

The results (see Table 3) showed that sense of place significantly predicted creativity (*β* = 0.68, *t* = 30.32, *p* < 0.001), and the prediction remained significant even when the mediating variable, psychological resilience, was included (*β* = 0.45, *t* = 20.66, *p* < 0.001). Sense of place was found to have a significant positive predictive effect on psychological resilience (*β* = 0.59, *t* = 20.64, *p* < 0.001); in addition, psychological resilience was found to have a significant positive predictive effect on creativity (*β* = 0.39, *t* = 23.86, *p* < 0.001). In addition, the lower and upper confidence intervals (95%) between the direct effect of sense of place on creativity and the mediating effect of psychological resilience were not zero (see Table 4). This suggests that, after controlling for age and grade, sense of place directly predicts creativity and sense of place indirectly predicts creativity through psychological resilience. The direct effect (0.45) and the mediating effect (0.23) accounted for 65.95 and 34.05% of the total effect, respectively. Figure 2 provides a graphic representation of these relationships.

**Table 3:** Mediation analysis results for the three variables.

Variables	Equation 1 (Outcome variables: Creativity)					Equation 2 (Outcome variables: Psychological resilience)					Equation 3 (Outcome variables: Creativity)				
	*β*	Boot SE	*t*	Boot 95% CI		*β*	Boot SE	*t*	Boot 95% CI		*β*	Boot SE	*t*	Boot 95% CI	
				Lower limit	Upper limit				Lower limit	Upper limit				Lower limit	Upper limit
Constant term	3.13	1.04	2.81^**^	1.07	5.14	36.72	1.57	25.52^***^	33.67	39.82	−11.11	1.06	−9.78^***^	−13.17	−8.98
Gender	−0.65 (−0.04)	0.33	−1.98	−1.30	−0.02	0.30 (0.02)	0.42	0.70	−0.53	1.10	−0.77 (−0.05)	0.29	−2.69^**^	−1.31	−0.19
Year	0.31 (0.061)	0.10	3.12^**^	0.11	0.50	0.33 (0.06)	0.13	2.63^**^	0.08	0.58	0.18 (0.04)	0.09	2.08^***^	0.00	0.35
Sense of place	0.68 (0.590)	0.02	30.32^***^	0.64	0.72	0.59 (0.45)	0.03	20.64^***^	0.53	0.66	0.45 (0.39)	0.03	20.66^***^	0.39	0.50
Psycho-logical resilience											0.39 (0.45)	0.02	23.86^***^	0.35	0.43
*R* ^2^	0.35					0.20					0.52				
F(df)	310.70*** (3,1707)					143.80^***^ (3,1707)					452.92^***^ (4,1706)				

** *p* < 0.01;

*** *p* < 0.001, all bilateral.

Gender 1 = male, gender 2 = female; year 1 = first year, year 2 = second year, year 3 = third year, year 4 = fourth year, year 5 = fifth year, and year 6 = sixth year.

**Table 4:** Total effect, direct effect, and indirect effect among variables.

	Effect size	Boot SE	Boot 95% CI		Relative effect size
			Lower limit	Upper limit	
Total effect	0.68	0.02	0.64	0.72	
Direct effect	0.45	0.03	0.39	0.50	65.95%
Indirect effect	0.23	0.02	0.19	0.27	34.05%

## Discussion

### Discussion of the Results

In this study, we developed a mediation model that investigated the relationship between sense of place and creativity among elementary school students during the COVID-19 pandemic. It was found that sense of place was positively related to creativity and to psychological resilience; also, psychological resilience was positively related to creativity, and psychological resilience mediated and buffered the direct effect of sense of place on creativity. These results are consistent with the hypotheses of this study and with previous research.

First, the findings are consistent with H1 and the existing literatures on the theory of creativity investment, indicating that sense of place is positively correlated to creativity. Creativity investment theory asserts that creative behavior is the result of the interaction between the individual and the environment. This result also confirms with the assertion in previous studies that positive environment has a facilitative effect on creativity (e.g., Richards, 1992; Ceylan et al., 2008; Pugsley and Acar, 2020) and that positive perception (or emotion) has a facilitative effect on creativity (e.g., Osofsky et al., 2011; Shenesey and Langhinrichsen-Rohling, 2015; Yawen et al., 2021). This finding implies that, during the COVID-19 pandemic, people may have been more creative if they had a stronger sense of local identity, local attachment, and local dependence. Just as creativity may be influenced by factors such as intelligence, knowledge, thinking style, personality traits, motivation, and environment, in accordance with creativity investment theory (Sternberg and Lubart, 1991), creativity is also a result of the interaction of abilities, processes, and environment (Plucker et al., 2004). In addition, different situations (e.g., Humke and Schaefer, 1996; Zhuravlyova and Zhuravlyov, 2015; Verneert et al., 2021) may produce types and levels creativity. Therefore, a person’s attachment to a particular environment and their identification with or even dependence on a particular place may affect creativity as such emotions strengthen or as the positive or negative environment around them evolves. On this basis, the stronger the identification, attachment, and dependence that elementary school students develop to the place they live in, the more likely they are creative in academic performance, especially in essay composition, artistic creations, and scientific inquiry activities related to the place they are familiar with. This type of creativity is exactly what was described earlier as Mini C and Little C. These creative things also have the potential to gradually develop students’ Pro C and even the Big C at some point in the future and are most likely related to students’ resilience during the COVID-19 pandemic. Second, the results of this study are also similar to the results of similar studies that have concluded that a positive environment positively influences psychological resilience (Xu et al., 2021) and that positive perceptions (or emotions) positively influence psychological resilience (Can, 2020). This result suggests that elementary school students’ sense of place may have enhanced their psychological resilience during the COVID-19 pandemic. One possible explanation is that they had developed identity with, attachment to, and even dependence on a specific place in response to a specific emotion, which in turn enhanced both internal and external protective factors. These protective factors may facilitate pupils’ recovery from negative experiences such as the novel coronavirus and their ability to adapt to their changing environment.

Third, these results are consistent with H3 and with studies that have showed that psychological resilience has a positive predictive effect on creativity (Xu et al., 2021). In other words, people with a high level of psychological resilience are more creative. This conclusion is consistent with psychological and sociological findings that reveal that when people are more psychologically resilient, individual problem-solving stamina and stress tolerance are stronger, which promotes creativity. Creativity is associated with the social component of psychological resilience because creative activity is a more direct expression of psychological resilience and is social in nature. In turn, psychological resilience is supported in the social environment and in social relationships that reinforce participation in creative activity. As mentioned in the theory of creativity investment, personality has a close relationship with creativity. This is mainly manifested in the resilience, willingness to take moderate risks, self-confidence, and courage to hold on to personal beliefs in the face of obstacles. It can be inferred that creativity is related to the social component of mental toughness. Because creative activity is a more direct expression of psychological resilience and is social in nature, in turn, psychological resilience is supported in the social environment and social relationships that reinforce the engagement in creative activities. Thus, social participation in creativity and psychological resilience are mutually supportive (Hertzog et al., 2008; McFadden and Basting, 2010; Xu et al., 2021). For elementary school students, a possible explanation for this outcome might be that their psychological resilience contributes to the creativity in their personality, which in turn facilitates the development of their creativity. As a result, students may achieve significant development in Mini C and Little C.

Fourth, these results corroborate H4 as well as those of previous studies. We found that psychological resilience can mediate between sense of place and creativity, acting as a pathway for sense of place to act on creativity. Neurologically, psychological resilience is seen to be related to emotional processing, and psychological resilience is also related to the functioning of different regions of the brain. Precisely, psychological resilience is positively correlated with the level of connectivity between the left orbitofrontal gyrus and the left inferior frontal gyrus, which enables flexible use of emotional resources and flexible control of interest processing, and with the level of connectivity of the right parahippocampal area, which promotes self-assessment (Shi et al., 2019; Xu et al., 2021). This gives highly resilient people the psychological ability to make the most of their emotional resources in the face of adversity or threat (Debbané et al., 2017; Xu et al., 2021), prompting individuals to challenge themselves in positive adaptation to adversity so that they can survive and thrive in their own positive adjustment (Sweetman et al., 2011; Xu et al., 2021). In other words, during negative experiences such as the COVID-19 pandemic, psychological resilience may have a moderating effect on readjustment to learning and life, prompt individuals to find better adaptation to a specific environment they already identify with, attach to, or even depend on after a mood disorder has occurred. At the same time, psychological resilience can also help individuals to more efficiently solve problems of daily life as well as to readjust to society and create new opportunities (Small-C creativity; Xu et al., 2021).

It is evident that, in the current study, psychological resilience partially moderates the relationship between sense of place and creativity. Data analysis showed that sense of place had the greatest impact on creativity (65.95%), which implies that the mediating role of psychological resilience is not dominant (only 34.05%). This also suggests that sense of place itself may have an important influence on creativity.

### Implications

This study has provided perspectives on the sense of place and creativity of elementary school students amid the COVID-19 pandemic. Our findings of the study have important theoretical and practical implications. In terms of its theoretical implications, this study is unique in linking sense of place to creativity, which deepens understanding of the impact of sense of place on creativity. Furthermore, the mediating and buffering role of psychological resilience demonstrated in this study suggests that elementary school students’ sense of place during the COVID-19 pandemic may have strengthened their psychological resilience, which in turn may have influenced their creativity. This actually enriches the theory of creativity investment as well. As for the practical implications, first, teachers need to highlight the sense of place among adolescents, so that students who identify with, attach to, and rely on a particular environment can become more creative. In addition, our research has found that psychological resilience has a buffering effect on the sense of place and creativity, suggesting that parents, teachers, and school administrators should focus on developing psychological resilience training for elementary school students to ensure that they will be able to cope with stressful events in future.

### Limitations and Future Directions

This study has some limitations. First, its design is cross-sectional. Second, all participants were from one elementary school, which may have affected the representativeness of the results. Future researchers could use a longitudinal study design to collect data over time, or they could look at participants from different schools or even from different sections of school populations. In addition, researchers could explore the relationship between sense of place, psychological resilience, and the various dimensions of creativity. Although the mechanisms by which sense of place affects creativity may be contested, this study provides empirical evidence that will be useful to future researchers.

## Conclusion

This study explored the relationship between sense of place and creativity and the role of psychological resilience in mitigating this relationship. The results indicated that sense of place was a positive predictor of creativity, i.e., elementary school students with a stronger sense of place tended to be more creative. Elementary school students with a strong sense of place were more psychologically resilient than those with a weaker sense of place. In addition, elementary school students who were more psychologically resilient were more creative than those who were less psychologically adaptive. Notably, most of the variance in creativity was attributable to sense of place, although psychological resilience did play a role, suggesting that a strong sense of place enhanced students’ creativity.

## Data Availability Statement

The raw data supporting the conclusions of this article will be made available by the authors, without undue reservation.

## Author Contributions

PL and YX designed the research. YX, QW, and DZ carried out the literature search and data analysis and wrote the paper. All authors have read and agreed to the submitted version of the manuscript.

## Funding

This work was supported by the Beijing Planning Office of Philosophy and Social Science (grant no. 18JDJYB010).

## Conflict of Interest

The authors declare that the research was conducted in the absence of any commercial or financial relationships that could be construed as a potential conflict of interest.

## Publisher’s Note

All claims expressed in this article are solely those of the authors and do not necessarily represent those of their affiliated organizations, or those of the publisher, the editors and the reviewers. Any product that may be evaluated in this article, or claim that may be made by its manufacturer, is not guaranteed or endorsed by the publisher.
